# P01.35. Antidepressant effects of Gastrodia elata Bl. at the genomic level

**DOI:** 10.1186/1472-6882-12-S1-P35

**Published:** 2012-06-12

**Authors:** L Sheen, E Chen

**Affiliations:** 1 Institute of Food Science & Technology, National Taiwan University, Taipei, Taiwan

## Purpose

Depression has been a serious issue as the annual worldwide suicide rate has been constantly increasing, according to the statistics published by WHO. In fact, WHO predicts that it will be the leading psychological disease by year 2020. Antidepressants in general, however, demonstrate serious side effects such as anxiety disorders, gastrointestinal problems, and sexual dysfunction in patients; therefore, it is important to find an effective way to prevent the occurrence of depression. Gastrodia eleta Bl., an Oriental herb that has been shown to demonstrate an anti-depression effect, was investigated in the form of water extraction.

## Methods

The water extract of Gastrodia eleta Bl. (WGE) was orally administered to Sprague-Dawley rats at the dose of 0.5 g/kg body weight each day for 21 consecutive days. Forced swimming test (FST) was performed to induce depression in the rodent model. Total mRNA samples were then obtained from the frontal cortex hippocampus and striatum, after having sacrificed the rats after 21 days. In order to deduce a possible anti-depression pathway at the genomic level, cDNA microarrays were performed to generate *in situ* gene expression profiles of depression relevant brain regions: cortex, hippocampus, and striatum. To confirm the findings, real-time polymerase chain reaction (QRT-PCR) analysis of several neuroplasticity-related, differentially expressed genes was performed.

## Results

The microarray data showed that WGE altered axongenesis/neurogenesis, nervous system development, and dopamine secretion pathways in cortex and hippocampus, from the evidence that they yield the lowest p-values from all other pathway matches with the KEGG database. Results from qRT-PCR validated that genes involved in neurogenesis, such as Map1b, RhoA, profilin-1, and CRMP2 were significantly altered (p<0.05) in the cortex and hippocampus.

## Conclusion

Neuroplasticity might be the mechanism by which WGE takes, and the results can serve as the basis of future antidepressant development, especially in the area of WGE demonstrating anti-depressant effects in the aspect of genomics.

